# Soft label collaborative view consistency enhancement with application to incomplete multi-view clustering

**DOI:** 10.1371/journal.pone.0326852

**Published:** 2025-07-01

**Authors:** Jie Zhang, Jiali Tang

**Affiliations:** School of Computer Engineering, Jiangsu University of Technology, Changzhou, China; Xinyu University, CHINA

## Abstract

Incomplete multi-view clustering (IMVC) is an unsupervised technique for clustering multi-view data when some view information is absent. However, most existing IMVC methods usually suffer from several significant challenges: (1) Inaccurate imputation or padding of missing data degrades clustering performance; (2) The ability to extract view features may decrease due to low-quality views, especially those that are inaccurately imputed. To overcome these challenges, in this paper, we introduce a novel IMVC framework, called soft label collaborative view consistency enhancement (SLC_CE). Firstly, we leverage the encoders of Transformers to construct a soft-label view information interaction module, which fully utilizes soft-labels to enhance view feature embeddings. Secondly, we employ soft labels to collaboratively impute missing features, addressing the incomplete multi-view data problem. Finally, we implement a consistency enhancement strategy across multi-level view features and soft labels to ensure high-quality feature extraction and imputation. Extensive experiments on several benchmark datasets demonstrate that the proposed SLC_CE method outperforms other state-of-the-art methods in real IMVC tasks.

## 1 Introduction

Multi-view clustering (MVC) is a well-known unsupervised learning technology that divides instances into clusters by utilizing their feature representations. These views can be derived from different sensors, domains, or feature extractors, providing a more comprehensive perspective of each instance [1–4]. The MVC technology [5–9] is fundamentally based on the assumption that all view data are fully available. However, in many real-world situations, multi-view data is frequently incomplete due to sensor malfunctions or missing information during collection. This poses significant challenges for directly applying MVC techniques to incomplete multi-view data.

To address this challenge, many incomplete multi-view clustering (IMVC) methods have been developed in recent years. Existing IMVC techniques [10–13] can be grouped into three main categories: matrix factorization-based IMVC, kernel learning-based IMVC, and graph learning-based IMVC. IMVC approaches based on matrix factorization [10,13–15] focus on decomposing multi-view data matrix to recover missing views and uncover shared representations. Wang *et al*. [16] fully explored spectral perturbation theory and then applies a tailored matrix completion approach to handle the similarity matrices of incomplete multi-view data. Rai *et al*. [15] adopted the non-negative matrix factorization (NMF) method to exploit the intrinsic geometric structure of the data distribution in each view. Kernel learning-based IMVC methods [11,17] cope with missing data by constructing a kernel matrix and then applying imputation techniques to estimate the missing values. For example, Liu *et al*. [17] integrated the imputation of incomplete kernel matrices with multiple kernel alignments to cluster in a unified framework. Graph-based methods [11,12,18] construct similarity graphs to represent relationships between data instances. This technique leverages the geometric structure of the graph to propagate information and handle missing data. Zhao *et al*. [12] employed unrestricted anchors to reconstruct relationships in high missing-rate data and integrated graph convolutional networks (GCNs) to obtain graph embeddings for clustering incomplete multi-view data. However, these aforementioned methods rely heavily on the quality of initial multi-view data and thus cannot fully capture the complex relationships between views.

Benefiting from the powerful feature representation capabilities of deep neural networks (DNNs), several deep IMVC methods [19,20,20–23] have been developed to deal with incomplete multi-view data. Autoencoder-based methods [24,25] use DNNs to learn feature representations and reconstruct missing views. Choudhury *et al*. [24] first imputed missing inputs using the *k*-nearest neighbor rule, and then preserved the structure of the input data in the latent space by incorporating Sammon’s stress as a regularizer in the objective function of the autoencoder. GAN-based deep IMVC methods [26–28] generate missing data through adversarial learning technology. Zhou *et al*. [26] employed adversarial learning and attention mechanisms to align latent feature distributions and quantify the importance of the modalities, respectively. With the development of contrastive learning methods, they have been integrated into the deep IMVC framework to learn consistent representations across views through contrastive learning strategies [29,30]. In [29], consistency learning is performed by maximizing mutual information between different views through contrastive learning, while missing views are recovered by minimizing conditional entropy through dual prediction. Despite the impressive progress of these methods, they still face issues with inaccurate imputation and low-quality feature extraction.

**Fig 1:**
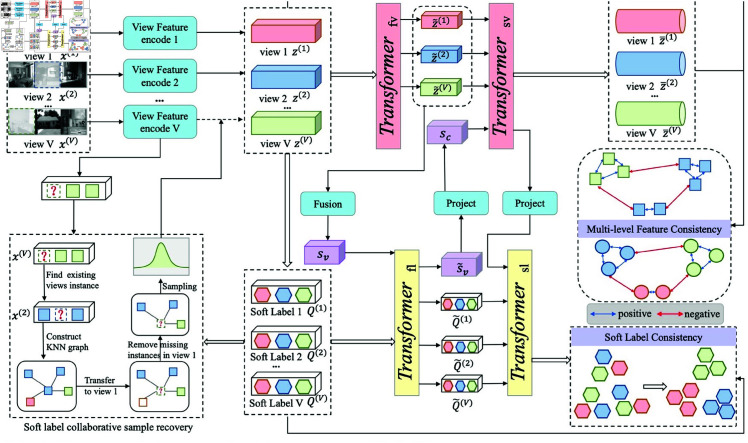
The overall framework of the proposed SLC_CE method.

To mitigate these limitations, we introduce a novel IMVC framework, called soft label collaborative view consistency enhancement (SLC_CE). As illustrated in Fig 1, the proposed SLC_CE method is designed to leverage the synergy between multiple views and soft labels, enabling accurate recovery of missing views. The proposed method designs an information interaction module by using soft-label information to enhance view feature embedding. In addition, to address incomplete multi-view data, we employ generated soft labels to recover missing view features using the *k*-nearest neighbor approach. Finally, to ensure the quality of view feature extraction and missing data recovery, we adopt a consistency enhancement strategy to constrain soft labels and multi-level view features. Extensive experimental results show the effectiveness of the proposed method in IMVC tasks.

The contributions of this work can be summarized as follows:

- We propose an information interaction module, which enriches view feature embeddings by utilizing soft labels. This effectively promotes interaction between views, thereby learning more robust feature representations. Meanwhile, our method uses soft-label information to collaboratively impute missing features across views, ensuring that the imputation process is guided by learned feature complementarity and consistency.
- We adopt a consistency enhancement strategy to constrain soft labels and multi-level view features. This helps maintain the quality of feature extraction and imputation and thus reduces the negative impact of low-confidence soft labels.
- Extensive experimental results on four incomplete multi-view datasets demonstrate the effectiveness and robustness of our proposed SLC_CE method compared to other state-of-the-art methods in complex IMVC tasks.

## 2 Related work

In this section, we briefly review related work on contrastive learning-based MVC, Transformer-based MVC, and IMVC methods.

### 2.1 Contrastive learning-based MVC

Contrastive learning is a well-established and effective unsupervised representation learning method, known for its ability to effectively generalize across different types of data representations [31–33]. Inspired by constrastive learning, contrastive multi-view learning has been proposed in the past few years [23,29,34]. For example, Tian *et al*. [35] applied contrastive learning to maximize mutual information between representations of different views, facilitating the learning of shared information across these views. Contrastive learning aims to increase the similarity between positive pairs of representations while minimizing the similarity between negative pairs, which closely aligns with clustering objectives. [36] used contrastive learning to align multi-view representations obtained from view-specific encoders, and then fused these aligned representations for single-view clustering. Moreover, Xu *et al*. [37] introduced an approach where multi-view representations are initially aligned using a parameter-shared network, and then contrastive learning is applied to ensure consistency between multi-view features and semantic labels. These contrastive multi-view learning methods highlight the flexibility of contrastive learning techniques in multi-view clustering models, providing a promising approach to improving both representation learning and clustering outcomes in multi-view scenarios.

Although contrastive learning has achieved notable progress in IMVC tasks, it still encounters several challenges, particularly those arising from feature distribution discrepancies and view misalignment. Due to the differences in the distribution of multi-view data, existing contrastive learning methods cannot effectively capture and align the shared information between different views. Additionally, these methods often emphasize learning single-view features while neglecting global consistency and precise alignment between views. This oversight may result in suboptimal performance when handling complex multi-view data.

### 2.2 Transformer-based MVC

Attention was first introduced in sequence-to-sequence tasks to help models focus on the most informative parts of the input representations. The Transformer architecture [38] fully relies on attention mechanisms, capturing global dependencies between input and output sequences. The Vision Transformer [39] extends the Transformer architecture to image classification by treating non-overlapping image patches of moderate size as input sequences, similar to the use of labels in translation tasks. Then hierarchical Transformers [40,41] introduce a novel technique using shifted image patch windows and variational patch segmentation strategies. They shift windows over non-overlapping patches to capture information from each patch combination, while variational patch segmentation (also known as patch merging) ensures that the learning model incorporates local regions into the broader image context.

Recently, Transformer has been applied to real IMVC task [22,42,43]. Its attention mechanisms establish associations across positions to capture global contextual features. Transformer-based IMVC methods can learn relationships between different views through attention mechanisms, thereby enhancing clustering performance. The attention mechanisms dynamically learn key features and interactions within each view. Additionally, the multi-head attention mechanisms further strengthen the modeling of relationships between different views, leading to more accurate clustering results. Therefore, we introduce the Transformer to enhance feature representation capabilities in this work.

### 2.3 Incomplete multi-view clustering (IMVC)

Incomplete multi-view clustering (IMVC) focuses on improving clustering performance in scenarios where multi-view data are incomplete. One widely used approach is to extract a shared subspace from incomplete data using matrix factorization. A seminal method, called partial multi-view clustering (PVC) [44], directly computes a common latent representation for complete instances while deriving view-specific latent representations for incomplete samples through matrix decomposition. Therefore, several matrix decomposition-based IMVC methods have been developed in recent years. For example, Rai *et al*. [15] proposed a graph-regularized non-negative matrix factorization method based on PVC. Hu *et al*. [45] proposed a doubly aligned incomplete multi-view clustering (DAIMC) method, which employs weighted semi-non-negative matrix factorization with *l*_2,1_ regularized regression to extract a shared representation. An alternative strategy in IMVC involves inferring missing samples. Wen *et al*. [46] developed a unified embedding alignment framework (UEAF) that addresses missing data by using an error matrix and reverse graph regularization to both complete the data and identify common structures. Then Wen *et al*. [47] explored high-order correlations across multiple views using tensor constraints, thereby learning similarity across multi-view graphs while recovering missing instances. A subspace clustering method has also been proposed to jointly perform data imputation and self-representation learning [48]. Inspired by generative adversarial networks (GANs) [49], Wang *et al*. [20] introduced a generative partial multi-view clustering approach that leverages GAN models to fill in missing data. More recently, [23] proposed an IMVC framework by combining consistency learning with data recovery. In addition, Lin *et al*. [29] presented a more generalized approach to learning representations from incomplete multi-view data.

Although these IMVC methods have demonstrated impressive performance, they often entail high computational costs and risk compromising data fidelity. The inherent complexity of feature extraction, alignment, and missing data inference across multiple views further hinders their scalability to large-scale datasets. Additionally, handling incomplete data can introduce noise or lead to the loss of important information, reducing data fidelity and impacting clustering performance. Therefore, preserving data integrity while improving computational efficiency poses a substantial challenge in IMVC applications.

## 3 Method

In this section, we introduce the proposed SLC_CE method for implementing IMVC tasks in detail.

### 3.1 Notations

Formally, let $X=\{X^{\left(v\right)}\in {\mathbb{R}}^{N\times d_{v}},v=1,2\ldots V\}$ represent the multi-view data, where *N* is the number of samples and $d_{v}$ is the feature dimensionality. Here, ${\text{X}}^{\left(\text{v}\right)}$ denotes the *v*-th view, and ′*NaN*′ represents missing instances. The parameter *K* is the cluster number.

### 3.2 Overall framework

Fig 1 illustrates the overall framework of the proposed SLC_CE method. First, the proposed model employs an information interaction Transformer to enable interactive learning between soft labels and view information. Therefore, it aims to fully utilize soft-label information to extract the features of multi-view data. To cope with incomplete data, we adopt soft-label information to collaborate with multi-view data using the *k*-nearest neighbor algorithm to generate the missing view features. Finally, to ensure the quality of view feature extraction and missing data recovery, we employ a consistency enforcement strategy to ensure the accuracy of generated soft labels and multi-level view features.

### 3.3 Information interaction transformer

As shown in Fig 1, we first learn the embedding of the multi-view data ${\text{X}}^{\left(\text{v}\right)}$. The features between different views are embedded into a common feature space. For a given sample $x_{i}^{\left(v\right)}\in {\mathbb{R}}^{d_{v}}$ from ${\text{X}}^{\left(\text{v}\right)}$, the embedding vector $z_{i}^{\left(v\right)}\in {\mathbb{R}}^{d_{e}}$ can be expressed as $z_{i}^{\left(v\right)}=Embedding(x_{i}^{\left(v\right)})$, where *d*_*e*_ represents the dimension of the embedding features. We then stack the embedding vectors to obtain the original multi-view embedding sequence $Z^{\left(v\right)}=[z_{i}^{\left(1\right)},z_{i}^{\left(2\right)},\ldots ,z_{i}^{\left(v\right)}]\in {\mathbb{R}}^{V\times d_{e}}$, which is further used as the input vector of the Transformer. Note that for incomplete multi-view data, we adopt the soft-label co-interpolation method (as detailed in Sect 3.4) to generate the embeddings of the missing views, ensuring that ${\text{Z}}^{\left(\text{v}\right)}$ is complete in all views. At the same time, the extracted view feature embedding $z_{i}^{\left(v\right)}$ is fed into the Transformer to enhance the view feature embedding. Therefore, we have

$$z_{i}^{\left(v\right)}=Embedding(x_{i}^{\left(v\right)})$$ (1)

$${\tilde{z}}_{i}^{\left(v\right)}=Transformer_{fv}(z_{i}^{\left(v\right)})$$ (2)

where Embedding(·) is a fully CNN and $Transformer_{fv}$ is the first layer of Transformer. $x_{i}^{\left(v\right)}$ is the incomplete multi-view data, and ${\tilde{z}}_{i}^{\left(v\right)}$ is the view feature embedding after *Transformer_fv_*. Here, an adaptive fusion layer is introduced to fuse the information from multiple views into a shared view feature $S_{v}$. The fusion process can be formulated as follows:

$$S_{v}=\sum_{v=1}^{V}\frac{z^{\theta_{v}^{\epsilon }}{\tilde{z}}_{v}}{\sum_{j}z^{\theta_{j}^{\epsilon }}}$$ (3)

where $\theta_{v}$ represents the learnable weight and ϵ is the adjustment factor. By interacting with the shared view feature $S_{v}$ to explore the correlations between the soft labels and the view embeddings, *Transformer*_*fl*_ attempts to obtain complementary information from the soft label. This process results in the enhanced soft-label $\tilde{Q}$ and feature embedding ${\tilde{S}}_{v}$ as follows:

$${\tilde{S}}_{v}$$ (4)

where [·,·] is the concatenation operation and $\tilde{Q}$ denotes enhanced cluster soft labels. Subsequently, the output features of *Transformer*_*fl*_ are propagated into the second layer.

The second layer is designed to extract high-level shared features, which is achieved by promoting the interaction and fusion between soft-label information and view features extracted from the first layer. Therefore, it obtains a more discriminative representation of the multi-view data. This layer incorporates two Transformer blocks, denoted as $Transformer_{sv}$ and *Transformer*_*sl*_. $Transformer_{sv}$ is used to enhance information across views and extract a high-level multi-view embedding ${\bar{z}}_{i}^{\left(v\right)}$. This is the enhanced representation of views by interacting with the shared soft label feature *S*_*c*_ and analyzing view correlations. Thus, we have

$$S_{c}=Proj_{vc}({\tilde{S}}_{v})$$ (5)

$$\left[{\bar{S}}_{c},{\bar{z}}_{i}^{\left(v\right)}]=Transformer_{sv}([S_{c},{\tilde{z}}_{i}^{\left(v\right)}]\right)$$ (6)

where $Proj_{vc}(\cdot )$ is a linear layer as a projection function designed to map vectors from the soft label feature space to the view feature space. Correspondingly, *Transformer*_*sl*_ is employed to complement information across soft labels and extract high-level soft label vectors $\bar{Q}$ by leveraging the shared feature $p_{v}$ and discerning soft label correlations, as illustrated as follows:

$$p_{v}=Proj_{cv}({\bar{S}}_{c})$$ (7)

$$\left[{\bar{p}}_{v},\bar{Q}]=Transformer_{sl}([p_{v},\tilde{Q}]\right)$$ (8)

where $Proj_{cv}(\cdot )$ is a linear layer as a projection function to map vectors from the view feature space to the soft label feature space. Through the propagation of vectors $S_{v}$, *S*_*c*_, and $P_{v}$ among the transformer blocks, we facilitate the sharing of information between the view and soft label feature spaces, thereby extracting more refined and effective features of views and soft labels.

### 3.4 Soft-label collaborative imputation

It is well known that when a partial sample of multi-view data is missing, we cannot effectively learn the embedded features. Most existing methods try to use existing views to complete the missing views to improve the feature extraction performance in the scenario where samples are missing. However, most of these methods only use the *k*-nearest neighbor algorithm for completion. Therefore, in this work, we make full use of the soft-label information to cooperate with the *k*-nearest neighbor method for completion and use the clustered soft-label vector *Q* to help generate the missing views. Specifically, for a sample *i*, let $ℰ=\{v|M_{i,v}=1\}$ represent the index of the existing view, and $ℳ=\{v|M_{i,v}=0\}$ represent the index of the missing view. To use the original multi-view embedding $z_{i}^{\left(v\right)}$ to supplement the missing features of the sample *i*, we first find the *k*-nearest neighbors in the projected soft label feature space. The neighbor set *D* can be constructed as follows:

$$𝒟=\{d|TopK(\sum_{j\in ℰ}\|{𝐳}_{j}-q_{d}{\|}_{2},d\in \{1,2,3,\ldots ,V\})\}$$ (9)

where TopK(·) is a function designed to identify the indices of the top *K* soft labels based on the smallest distance between embedding vectors and soft label vectors. Then, we employ a statistical method to describe the distribution of the missing views. We assume that the missing views $\{{𝐞}_{m}{\}}_{m\in ℳ}$ satisfy the multivariate Gaussian distribution 𝒩(μ,Σ), whose mean vector and covariance matrix are denoted as follows:

$$\mu =\frac{\sum_{d\in 𝒟}q_{d}}{\left|𝒟\right|}$$ (10)

$$\Sigma =\frac{1}{|𝒟|-1}\sum_{d\in 𝒟}(q_{d}-\mu )(q_{d}-\mu {)}^{T}$$ (11)

For the missing views, we sample from this distribution |ℳ| times and substitute the missing views with the sampled results. Consequently, we can obtain the complete embeddings for the incomplete multi-view data. By reconstructing the missing multi-view data, our proposed method further enhances its performance in incomplete information clustering.

### 3.5 Soft-label and view consistency enhancement

Using the aforementioned soft-label view information interaction Transformer, we extract two multi-view embeddings $z_{i}^{\left(v\right)}$ and ${\bar{z}}_{i}^{\left(v\right)}$ from different layers, respectively. To enable our encoder to effectively extract the features, it is crucial to enhance the discriminative ability of these embeddings. Specifically, according to the consistency between multiple views, the embedded features of samples from different views should be aligned. In addition, we can fully utilize the consistent features of multi-view data to improve the discriminative ability of $z_{i}^{\left(v\right)}$ and ${\bar{z}}_{i}^{\left(v\right)}$. Taking these factors into consideration, we introduce the embedding enhancement of multi-level view features. To learn more effective embeddings $z_{i}^{\left(v\right)}$ and ${\bar{z}}_{i}^{\left(v\right)}$, we use contrastive learning to align the embeddings of the same sample from different views. Therefore, we employ the loss function in the proposed model as follows:

$${ℒ}^{\left(z^{\left(m\right)},z^{\left(n\right)}\right)}=-\log\frac{e^{s\left(z_{i}^{\left(m\right)},z_{i}^{\left(n\right)}\right)/\tau }}{\sum_{j=1}^{N}\left[e^{s\left(z_{i}^{\left(m\right)},z_{j}^{\left(m\right)}\right)/\tau }+e^{s\left(z_{i}^{\left(m\right)},z_{j}^{\left(n\right)}\right)/\tau }\right]},$$ (12)

$${ℒ}^{\left({\bar{z}}^{\left(m\right)},{\bar{z}}^{\left(n\right)}\right)}=-\log\frac{e^{s\left({\bar{z}}_{i}^{\left(m\right)},{\bar{z}}_{i}^{\left(n\right)}\right)/\tau }}{\sum_{j=1}^{N}\left[e^{s\left({\bar{z}}_{i}^{\left(m\right)},{\bar{z}}_{j}^{\left(m\right)}\right)/\tau }+e^{s\left({\bar{z}}_{i}^{\left(m\right)},{\bar{z}}_{j}^{\left(n\right)}\right)/\tau }\right]},$$ (13)

$${ℒ}_{con}={ℒ}^{\left(z^{\left(m\right)},z^{\left(n\right)}\right)}+{ℒ}^{\left({\bar{z}}^{\left(m\right)},{\bar{z}}^{\left(n\right)}\right)}.$$ (14)

where *m* and *n* refer to the indices of the *m*-th and *n*-th views, respectively. s(·,·) represents the cosine similarity and τ is the temperature parameter.

As previously mentioned, we utilize clustering soft labels to assist in completing missing data. This means that the quality of the recovery data depends largely on the accuracy of the soft labels. Here, we adopt contrastive learning to optimize the soft clustering process. For the *m*-th view, *Q*^*m*^(:,*j*) have (*Vk*–1) pairs, where the (*V*–1) pairs $\{Q^{m}(:,j),Q^{n}(:,j){\}}_{m\neq n}$ are positive and the rest *V*(*k*–1) pairs are negative. Thereby, the contrastive loss can be defined as follows:

$${ℒ}_{Q}^{mn}=-\frac{1}{k}\sum_{j=1}^{k}\log\frac{e^{d(Q^{m}(:,j),Q^{n}(:,j))/\tau }}{\sum_{k\prime =1}^{k}\sum_{\nu =m,n}e^{d(Q^{m}(:,j),Q^{\nu }(:,k\prime ))/\tau }-e^{1/\tau }},$$ (15)

Similarly, our refined soft label feature consistency enhancement optimization is as follows:

$${ℒ}_{\bar{Q}}^{mn}=-\frac{1}{k}\sum_{j=1}^{k}\log\frac{e^{d({\bar{Q}}^{m}(:,j),{\bar{Q}}^{n}(:,j))/\tau }}{\sum_{k\prime =1}^{k}\sum_{\nu =m,n}e^{d({\bar{Q}}^{m}(:,j),{\bar{Q}}^{\nu }(:,k\prime ))/\tau }-e^{1/\tau }},$$ (16)

where d(·,·) represents the cosine distance to measure the similarity between two labels, and τ is the temperature parameter. Moreover, we use the cross entropy as a regularization term to avoid the samples being assigned into a single cluster. Thus, the label consistency learning is formulated as follows:

$${ℒ}_{Q}=\frac{1}{2}\sum_{m=1}^{V}\sum_{n\neq m}L_{Q}^{mn}+\frac{1}{2}\sum_{m=1}^{V}\sum_{n\neq m}L_{\bar{Q}}^{mn}+\sum_{m=1}^{V}\sum_{j=1}^{k}s_{j}^{m}\log s_{j}^{m},$$ (17)

where $s_{j}^{m}=\frac{1}{N}\sum_{i=1}^{N}q_{ij}^{m}$. After fine-tuning the labels through contrastive learning, the similarity between positive pairs is increased, resulting in latent features with a more distinct clustering structure.

Therefore, the full loss function of the proposed method is given as follows:

$$ℒ=\alpha *{ℒ}_{con}+\beta *{ℒ}_{Q}.$$ (18)

In this paper, the optimization of the objective function shown in Eq 18 is an end-to-end learning process. The total training process of the proposed model is summarized in Algorithm 1.

**Algorithm 1. The proposed SLC_CE algorithm.**



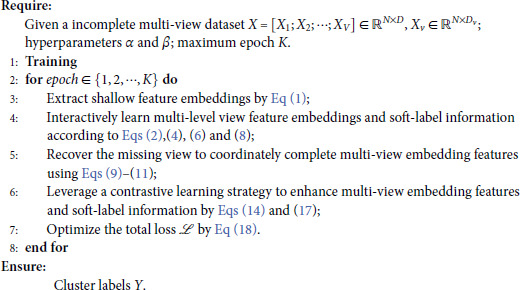



## 4 Experimental results and analysis

### 4.1 Datasets and metrics

We conducted experiments on four benchmark multi-view datasets: Aloi-100, Scene15, MNISTUSPS, and NoisyMNIST, as summarized in Table 1. To evaluate the robustness of our proposed method, we assessed the clustering performance of the proposed method under different missing rates, specifically [0.1, 0.3, 0.5, 0.7], across all datasets. The clustering performance was measured using three widely used clustering metrics: accuracy (ACC), normalized mutual information (NMI), and adjusted Rand index (ARI). Generally speaking, higher values for these indicators correspond to better clustering performance.

**Table 1:** The descriptions of four benchmark multi-view datasets.

Dataset	Samples	Views	Classes	Dimension
Aloi-100	10,800	3	100	4096/2048
Scene15	4,485	3	15	20/59
MNISTUSPS	5,000	2	10	784/784
NoisyMNIST	30,000	2	10	784/784

### 4.2 Comparison methods

In this experiment, we evaluated the proposed SLC_CE method against nine state-of-the-art IMVC techniques: **COMPLETER** [23] addresses missing views by minimizing the conditional entropy between different views through dual prediction. **DCP** [29] develops a unified framework to learn consistent representations across views and recover missing views in incomplete multi-view representation learning. **CBG** [50] proposes a flexible and efficient incomplete large-scale multi-view clustering method based on a bipartite graph framework to solve the problems of high complexity and expensive time consumption. **CPSPAN** [51] employs pair-observed data alignment to guide the construction of instance-to-instance correspondences across views. **PIMVC** [52] proposes a novel graph-regularized projective consensus representation learning model for IMVC. **APADC** [53] introduces an imputation-free deep IMVC method that incorporates distribution alignment in feature learning. **DIVIDE** [54] utilizes random walks to identify data pairs on a global scale, rather than locally, effectively reducing false negatives in contrastive learning. **SCSL** [55] proposes a sample-level cross-view similarity learning (SCSL) method for IMVC. **DVIMC** [56] introduces a variational autoencoder-based method to address the missing data problem in IMVC. VITAL [57] learns both common and specific information by modeling each sample as a Gaussian distribution. It uses variational inference for contrastive learning across views.

### 4.3 Implementation details

We employed a multi-layer perceptron (MLP) with a fully connected (Fc) network as the encoder to extract the features. For each view, the encoder structure was set as follows: *Input*–*Fc*500–*Fc*2000–*Fc*2000–*Fc*10. The temperature parameter τ was fixed at 1 for all experiments. We used the Adam optimizer with a learning rate of 1.0e-4. Due to differences in the distributions of the datasets, the hyperparameters were adjusted accordingly. For the Aloi-100 dataset, we used a batch size of 512, trained for 200 epochs, and set α to 0.1 and β to 1. For the Scene15 dataset, we used a batch size of 256, trained for 200 epochs, and set α to 0.01 and β to 1. For the MNIST-USPS dataset, we used a batch size of 512, trained for 200 epochs, and set *α* to 0.1 and β to 1. For the NoisyMNIST dataset, we used a batch size of 1024, trained for 200 epochs, with α set to 0.01 and β to 1. All experiments were carried out on an Ubuntu system with an NVIDIA GeForce RTX 3090 GPU (24.0 GB memory).

### 4.4 Experimental results

To evaluate the performance of our proposed SLC_CE method in IMVC tasks, we compared it with several state-of-the-art methods. Table 2 presents the clustering results of our SLC_CE method and the baseline models on four incomplete datasets. The best results are highlighted in bold, and the second-best results are underlined. From the experimental results, we can get the following observations:

**Table 2:** The clustering performances of various IMVC methods with different missing rate settings. The best and second-best results are highlighted in bold and underlined, respectively. The symbol ‘N/A’ indicates a memory overflow error.

η	Methods	Aloi-100			Scene15			MNISTUSPS			NoisyMNIST		
		ACC	NMI	ARI	ACC	NMI	ARI	ACC	NMI	ARI	ACC	NMI	ARI
0.1	COMPLETER(2021)	64.1	92.1	64.6	43.6	46.0	26.7	96.7	92.9	90.3	93.0	88.8	86.8
	DCP(2022)	67.4	90.7	65.8	42.4	43.6	23.7	97.5	92.3	90.0	93.3	91.2	87.5
	CBG(2022)	86.7	97.2	83.5	30.9	31.9	14.3	98.1	93.0	93.0	95.9	90.3	95.5
	CPSPAN(2023)	77.7	93.9	74.4	45.2	44.9	29.9	80.3	79.4	72.2	62.3	63.3	52.4
	PIMVC(2023)	84.7	98.5	85.3	36.2	37.7	21.5	98.1	96.3	96.9	N/A	N/A	N/A
	APADC(2023)	81.9	93.5	72.5	47.1	48.1	27.7	98.9	95.9	97.4	98.3	93.9	97.1
	DIVIDE(2024)	91.4	99.0	93.7	45.2	45.4	31.0	98.3	95.6	96.3	97.3	95.3	95.4
	SCSL(2024)	93.1	98.0	93.4	32.8	28.9	14.6	55.9	44.3	60.0	N/A	N/A	N/A
	DVIMC(2024)	87.8	92.3	85.5	48.8	48.1	30.5	87.3	85.0	81.2	98.5	95.7	96.7
	VITAL(2024)	91.7	98.7	91.5	41.9	45.3	27.2	98.8	**98.5**	95.2	97.2	94.1	96.2
	SLC_CE(Our)	**96.8**	**99.8**	**96.8**	**49.0**	**49.3**	**31.4**	**99.8**	97.7	**98.4**	**99.8**	**97.4**	**98.4**
0.3	COMPLETER(2021)	63.9	91.5	62.2	41.5	44.1	23.7	91.1	87.1	85.3	92.3	87.7	86.3
	DCP(2022)	61.6	88.4	61.7	38.4	42.5	23.5	93.2	90.0	86.8	92.5	88.3	86.6
	CBG(2022)	83.4	96.5	82.7	28.4	27.5	11.7	95.3	91.7	92.1	95.3	89.3	92.3
	CPSPAN(2023)	73.6	92.6	68.8	42.7	42.1	26.8	75.1	75.5	65.3	61.8	62.2	52.1
	PIMVC(2023)	83.3	95.8	83.7	33.5	33.2	17.9	97.2	94.7	94.8	N/A	N/A	N/A
	APADC(2023)	77.9	92.9	73.3	41.1	43.9	22.9	97.1	93.4	95.0	95.2	91.1	91.4
	DIVIDE(2024)	89.2	97.9	91.5	44.7	44.2	29.3	97.3	94.6	95.3	96.7	94.0	95.0
	SCSL(2024)	90.9	97.2	91.3	31.2	27.9	13.3	54.4	42.2	57.7	N/A	N/A	N/A
	DVIMC(2024)	85.9	89.6	81.1	47.5	48.0	30.1	84.9	82.8	77.0	96.5	94.7	95.6
	VITAL(2024)	89.7	97.9	90.0	41.5	43.3	26.8	97.2	94.3	93.1	96.3	92.5	93.2
	SLC_CE(Our)	**96.2**	**98.7**	**96.1**	**48.5**	**48.6**	**30.8**	**99.6**	**97.1**	**97.5**	**98.8**	**96.5**	**97.6**
0.5	COMPLETER(2021)	61.4	89.3	60.5	38.7	41.2	21.6	89.8	85.3	83.5	90.0	84.9	83.1
	DCP(2022)	59.8	87.6	61.1	37.4	41.5	23.1	91.5	89.2	85.0	92.2	88.0	85.9
	CBG(2022)	82.7	95.7	81.6	26.4	25.9	10.2	92.2	89.1	91.5	94.4	88.9	91.8
	CPSPAN(2023)	73.1	90.4	67.5	42.5	41.3	25.2	74.8	75.3	64.7	60.4	61.7	50.1
	PIMVC(2023)	82.2	93.1	82.7	32.5	33.1	16.7	97.0	93.8	93.9	N/A	N/A	N/A
	APADC(2023)	77.5	90.4	72.1	40.8	43.1	22.3	96.2	92.6	94.1	94.3	90.5	90.7
	DIVIDE(2024)	88.4	96.7	88.9	44.2	46.6	29.0	96.9	94.4	95.2	94.4	91.7	92.9
	SCSL(2024)	90.8	96.9	91.2	31.6	29.3	14.9	53.8	39.3	55.1	N/A	N/A	N/A
	DVIMC(2024)	85.8	88.2	80.8	46.1	46.5	30.4	83.6	82.3	76.5	95.6	91.4	90.7
	VITAL(2024)	87.2	97.5	88.5	41.1	41.8	25.3	94.3	86.1	88.1	95.6	89.1	90.8
	SLC_CE(Our)	**94.9**	**97.8**	**95.7**	**47.8**	**48.2**	**30.6**	**98.0**	**96.1**	**97.4**	**97.5**	**95.7**	**96.6**
0.7	COMPLETER(2021)	37.5	75.7	32.5	35.6	38.5	20.5	74.5	72.5	58.0	73.3	66.0	59.9
	DCP(2022)	50.9	82.9	51.2	36.8	39.2	21.1	72.7	72.6	61.1	74.1	67.2	60.6
	CBG(2022)	83.5	95.2	81.8	24.9	24.8	8.8	89.0	80.2	77.4	77.6	68.9	64.4
	CPSPAN(2023)	72.8	89.7	66.5	38.8	38.7	21.6	67.4	67.7	55.6	57.5	60.1	46.2
	PIMVC(2023)	80.4	92.4	80.6	31.8	32.1	16.3	89.5	80.9	79.1	N/A	N/A	N/A
	APADC(2023)	76.2	90.1	72.0	38.7	40.7	22.2	90.7	82.0	81.2	79.4	74.5	69.6
	DIVIDE(2024)	87.8	96.0	88.3	42.2	40.6	24.9	90.9	81.7	81.9	88.1	77.7	77.4
	SCSL(2024)	52.3	85.5	40.3	30.9	28.6	14.2	52.6	38.2	53.7	N/A	N/A	N/A
	DVIMC(2024)	84.1	80.3	79.5	39.8	38.8	24.4	82.7	81.8	75.5	91.2	82.4	82.7
	VITAL(2024)	85.5	96.1	86.9	40.7	40.8	24.8	91.6	83.3	84.3	91.9	83.5	84.6
	SLC_CE(Our)	**93.8**	**97.5**	**93.2**	**43.9**	**42.3**	**25.8**	**92.1**	**83.9**	**84.4**	**92.5**	**83.7**	**85.2**
0.9	COMPLETER(2021)	25.4	60.3	25.3	30.4	33.1	16.8	65.4	63.8	51.5	63.1	60.6	48.8
	DCP(2022)	45.6	71.9	43.5	30.8	32.5	15.6	63.4	64.3	55.4	66.6	59.1	51.4
	CBG(2022)	71.1	85.6	71.9	20.8	19.6	5.2	80.5	72.3	69.1	68.9	61.4	55.5
	CPSPAN(2023)	65.6	78.4	55.8	30.4	29.9	17.4	60.5	61.3	50.7	50.4	53.6	41.8
	PIMVC(2023)	71.8	83.5	72.2	25.6	27.7	10.5	78.5	71.3	70.1	N/A	N/A	N/A
	APADC(2023)	70.7	82.3	64.3	30.6	31.8	14.3	79.8	71.6	73.2	70.1	63.8	60.4
	DIVIDE(2024)	79.3	88.8	76.6	35.6	34.8	17.9	82.7	76.6	78.1	80.4	73.5	74.6
	SCSL(2024)	46.8	77.7	36.4	22.8	20.4	9.3	44.3	30.5	45.5	N/A	N/A	N/A
	DVIMC(2024)	80.3	74.3	73.8	31.7	33.3	19.8	78.5	76.8	70.4	86.6	77.3	75.4
	VITAL(2024)	81.3	90.7	78.7	37.7	36.6	20.5	81.5	76.1	83.3	88.1	78.2	78.8
	SLC_CE(Our)	**84.7**	**91.5**	**83.9**	**38.8**	**37.6**	**21.3**	**83.9**	**78.8**	**79.6**	**89.4**	**79.1**	**80.5**

1) It can be observed that our method outperforms other competitors, such as CBG, PIMVC, and SCSL. Traditional IMVC methods often rely on shallow learning models to process multi-view data, which limits their ability to capture nonlinear relationships and higher-order features. Most existing methods attempt to fill in missing views by leveraging available views, primarily using *k*-nearest neighbor (KNN) algorithms to complete the missing data and improve feature extraction. However, these methods struggle to fully capture the complex structural information inherent in multi-view data. In contrast, our method combines soft-label information with KNN for data completion and employs a clustered soft-label vector *Q* to recover the missing views. This allows our approach to more effectively handle complex real-world scenarios. The information interaction module leverages soft labels to enhance the feature embeddings across views, improving inter-view interactions and learning more robust feature representations. They ultimately lead to superior clustering performance, demonstrating the effectiveness of our soft-label imputation strategy.
2) Different other state-of-the-art deep IMVC approaches such as CPSPSN, DCP, and APADC, which predict missing views but do not fully leverage label information, our approach uses soft labels to fill in missing features across views more effectively. This is due to the guidance of learned feature relationships and consistency. This strategy significantly boosts the model’s performance and enhances its capability to handle missing data effectively.
3) We can observe from the results that our approach surpasses IMVC methods such as DIVIDE and COMPLETER, which also employ contrastive learning strategies to enhance view consistency. In contrast, our approach leverages a multi-level contrastive learning strategy to enforce consistency between soft labels and multi-level view features. This strategy not only preserves the quality of feature extraction and imputation, but also mitigates the negative effects of low-confidence soft-labels, resulting in more robust performance.

### 4.5 Ablation study

In this subsection, we evaluated the contribution of each component in our method with the same experimental setting. Specifically, we constructed three variants of the proposed method: (A) excluding the soft-label and view consistency enforcement part, called SV_CE (w/o SV_CE); (B) removing the soft-label view interaction Transformer in the graph and replacing it with Multi-Layer Perceptron (MLP), referred to as SV_IT (w/o SV_IT); (C) eliminating the soft-label collaborative part in the missing value recovery process, called SLC (w/o SLC). Table 3 shows the ablation results of our proposed method on four different datasets. It can be seen that removing any component from our method or replacing our proposed module with an alternative module significantly degrades the clustering performance. This shows that each component of our proposed method plays a vital role in IMVC tasks. Specifically, our SV_CE (w/o SV_CE) component performs consistency feature alignment operations from the view features and soft clustering levels through a contrastive learning strategy to learn feature consistency more effectively. This helps to reduce the negative impact of low-confidence soft labels and maintain the quality of feature extraction and filling. The SV_IT (w/o SV_IT) component plays a key role during the feature extraction. We flexibly employ the attention mechanism to interactively learn view features, and use soft clustering to maximize the utilization of soft labels, thereby enriching the view feature embedding. This effectively promotes the interaction between views and thus learns more powerful feature representations. The SLC (w/o SLC) component incorporates soft label information to guide the restoration of missing values, ensuring that the model accurately restores missing samples.

**Table 3:** Ablation experiments of our SLC_CE method with a missing rate of 0.7 on four datasets.

Method	Aloi-100			Scene15			MNISTUSPS			NoisyMNIST		
	ACC	NMI	ARI	ACC	NMI	ARI	ACC	NMI	ARI	ACC	NMI	ARI
SLC_CE (w/o SV_CE)	56.3	79.3	40.7	25.5	23.6	9.4	54.4	55.9	36.9	58.5	56.2	36.9
SLC_CE (w/o SV_IT)	92.5	97.1	92.0	41.4	41.1	24.1	91.9	83.5	84.0	91.7	82.2	83.7
SLC_CE (w/o SLC)	93.5	97.3	92.9	42.1	42.2	25.4	90.9	82.2	82.0	92.1	83.3	84.8
SLC_CE (Our)	**93.8**	**97.5**	**93.2**	**43.9**	**42.3**	**25.8**	**92.1**	**83.9**	**84.4**	**92.5**	**83.7**	**85.2**

### 4.6 Convergence analysis

**Fig 2:**
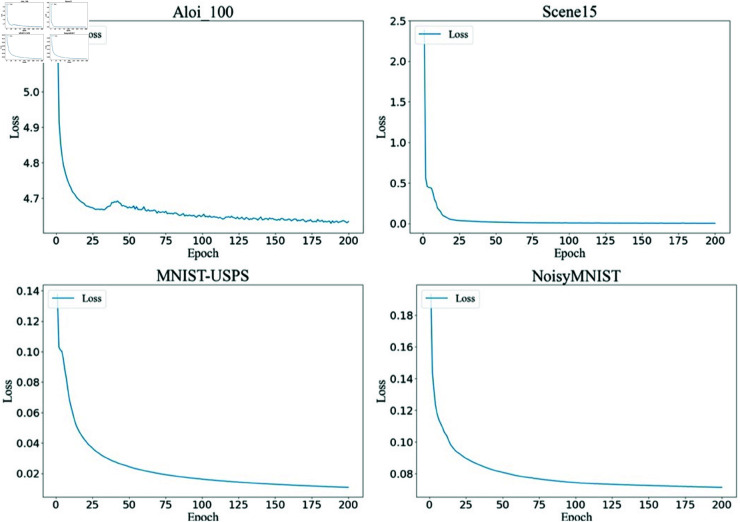
The loss values of the proposed SLC_CE method on the four datasets.

In this subsection, we conducted a convergence analysis experiment on four benchmark datasets. Fig 2 illustrates the convergence of the proposed SLC_CE method on different multi-view datasets, each with a missing rate of 0.7. It can be seen that the loss decreases quickly in the first 50 epochs, then continues to decline gradually with minor fluctuations before eventually stabilizing. These convergence results demonstrate the reliability and effectiveness of the proposed method in tackling the incomplete multi-view clustering (IMVC) problem, demonstrating its consistent performance even under challenging conditions.

### 4.7 Parameter analysis

**Fig 3:**
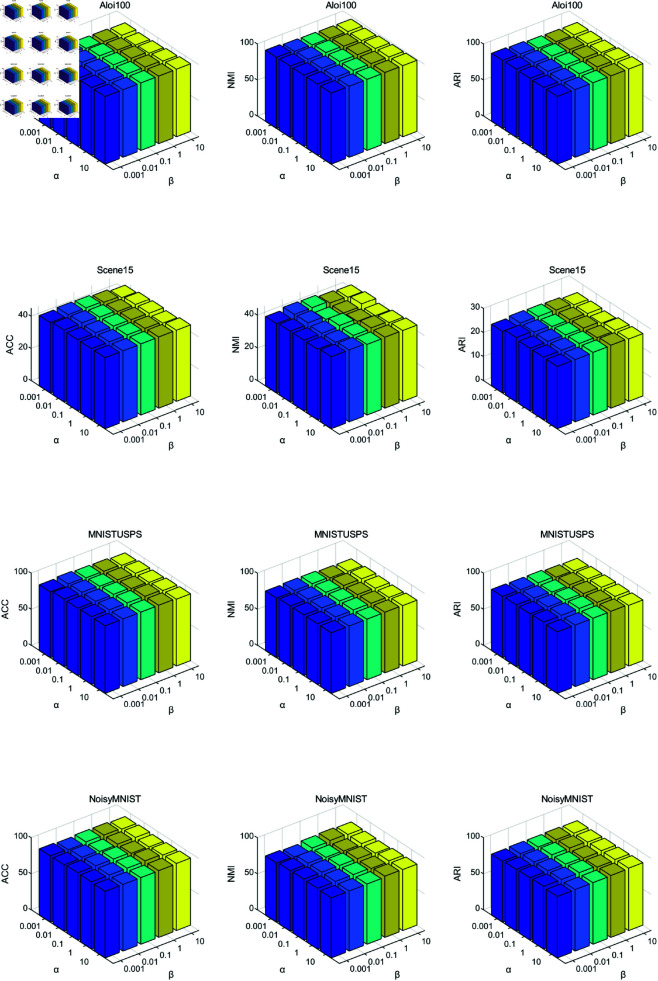
The parameter analysis of the proposed method on four datasets.

In this subsection, we conducted experiments on four datasets to evaluate the parameter sensitivity of the proposed method. Here, we set the missing rate to 0.7 in this experiment. The proposed model includes two trade-off coefficients, α and β in Eq 18, with values ranging from 10^−3^ to 10. Fig 3 shows the experimental results of our proposed method on four incomplete multi-view datasets. The results indicate that our method maintains stable clustering performances across a wide range of parameters, demonstrating the insensitivity of our proposed method under different real applications.

### 4.8 Visualization

**Fig 4:**
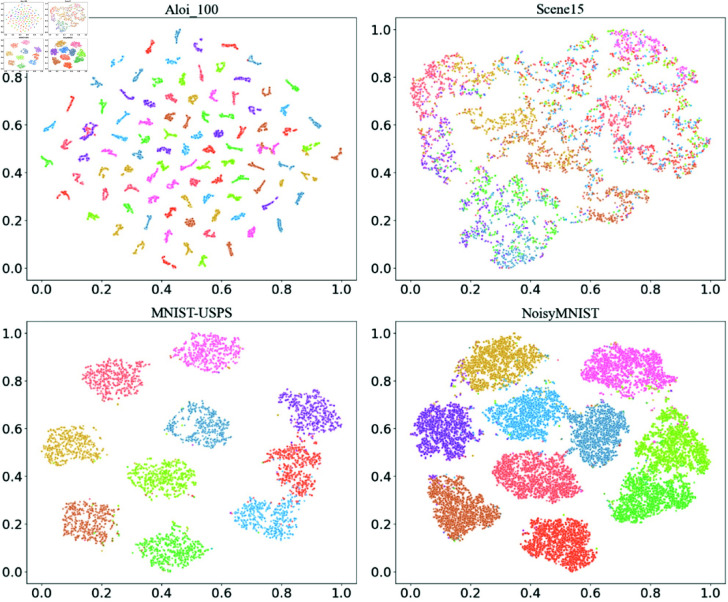
The visualization results of the proposed SLC_CE method on the four datasets.

To intuitively assess the effectiveness of the proposed SLC_CE model, we employed the *t*-SNE algorithm to visualize the distribution of latent features learned by the model with a missingness rate of 0.7. As illustrated in Fig 4, the generated clusters are distinctly separated with clear boundaries, demonstrating that our method effectively captures meaningful features from the multi-data. The clarity of these clustering results further confirms the robustness and effectiveness of the proposed method in handling complex clustering tasks.

### 4.9 Complexity analysis

In this subsection, we evaluate the computational efficiency of our method by measuring the number of parameters, running time, and floating point operations (FLOPs), and compare it with several state-of-the-art deep incomplete multi-view clustering approaches. The results in Table 4 show that our method outperforms other IMVC methods regarding the number of parameters, running time, and FLOPs. It highlights that the proposed model outperforms other state-of-the-art methods in clustering accuracy and maintains competitive computational efficiency, thus improving its overall effectiveness and scalability.

**Table 4:** Complexity analysis of competing deep IMVC methods on the NoisyMNIST dataset.

Method	FLOPs	Time (s)	Parameters
	(Millions)	(Seconds)	(Millions)
APADC (2023)	5.3	1321	9.7
DIVIDE (2024)	7.6	1982	17.2
DVIMC (2024)	6.8	1773	14.6
SLC_CE (Our)	6.7	1680	12.5

## 5 Conclusion

In this paper, we introduce a soft label collaborative view consistency enhancement (SLC_CE) method for IMVC. Our approach leverages a soft-label view information interaction Transformer to fully exploit soft-label information for enhancing view feature embeddings. To handle the challenge of incomplete multi-view data, we employ the *k*-nearest neighbor method, guided by soft-label information, to recover missing view features across views. Additionally, we incorporate a consistency enhancement strategy to ensure accurate view feature extraction and missing data recovery by constraining soft labels and multi-level view features. Extensive experimental results have demonstrated that our SLC_CE method outperforms other state-of-the-art methods in clustering tasks involving incomplete multi-view data.

Although the proposed method achieves satisfactory clustering performance, it has several limitations. Specifically, it employs traditional autoencoders as the backbone network, which limits its feature extraction capability. Therefore, we will incorporate a more powerful feature extraction model, such as multimodal vision-language models, to enhance multi-view feature representations. In addition, the semi-paired problem in multi-view data is common in many applications, and adapting the proposed method to handle it remains a significant challenge.
